# Prenatal care in the Brazilian public health services

**DOI:** 10.11606/s1518-8787.2020054001458

**Published:** 2020-01-14

**Authors:** Maria do Carmo Leal, Ana Paula Esteves-Pereira, Elaine Fernandes Viellas, Rosa Maria Soares Madeira Domingues, Silvana Granado Nogueira da Gama

**Affiliations:** I Fundação Oswaldo Cruz. Escola Nacional de Saúde Pública (ENSP/Fiocruz). Departamento de Epidemiologia e Métodos Quantitativos em Saúde (DEMQS). Rio de Janeiro, RJ, Brasil; II Fundação Oswaldo Cruz. Instituto Nacional de Infectologia Evandro Chagas. Laboratório de Pesquisa Clínica em HIV/Aids (INI/Fiocruz). Rio de Janeiro, RJ, Brasil

**Keywords:** Prenatal Care, Maternal-Child Health Services, Health Care Quality, Access, and Evaluation, Socioeconomic Factors, Health Status Disparities

## Abstract

**OBJECTIVE:**

To verify regional inequalities regarding access and quality of prenatal and birth care in Brazilian public health services and associated perinatal outcomes

**METHODS:**

Birth in Brazil was a national hospital-based survey conducted between 2011 and 2012, which included 19,117 women with public-funded births. Regional differences in socio-demographic and obstetric characteristics, as well as differences in access and quality of prenatal and birth care were tested by the χ^2^ test. The following outcomes were assessed: spontaneous preterm birth, provider-initiated preterm birth, low birth weight, intrauterine growth restriction, Apgar in the 5th min < 8, neonatal and maternal near miss. Multiple and non-conditional logistic regressions were used for the analysis of the associated perinatal outcomes, with the results expressed in adjusted odds ratio and 95% confidence interval.

**RESULTS:**

Regional inequalities regarding access and quality of prenatal and birth care among users of public services are still evident in Brazil. Pilgrimage for birth associated with all perinatal outcomes studied, except for intrauterine growth restriction. The odds ratios ranged between 1.48 (95%CI 1.23–1.78) for neonatal near miss and 1.62 (95%CI 1.27–2.06) for provider-initiated preterm birth. Among women with clinical or obstetric complications, pilgrimage for birth associated with provider-initiated preterm birth and with Apgar in the 5th min < 8, odds ratio of 1.98 (95%CI 1.49–2.65) and 2.19 (95%CI 1.31–3.68), respectively. Inadequacy of prenatal care associated with spontaneous preterm birth in both groups of women, with or without clinical or obstetric complications.

**CONCLUSION:**

Improvements in the quality of prenatal care, appropriate coordination and comprehensive care at the time of birth have a potential to reduce prematurity rates and, consequently, infant morbidity and mortality rates in the country.

## INTRODUCTION

Prenatal care is a set of simultaneously preventive, health promotion, diagnostic and curative actions targeting favorable pregnancy outcomes for women and their children^1^.

The Brazilian recommendation for prenatal care in 2012 was of at least six prenatal care visits, including vaccination, routine diagnostic laboratory tests, and the use of supplements or medical treatment for complications^2^. All the procedures should be registered in the hand-held prenatal notes, aiming reference and counter-reference at the time of birth. The bond between the pregnant woman and the place of birth is also recommended to prevent pilgrimage: the search for hospital care during labor^3^.

Data from the Brazilian Live Birth Information System (SINASC) show the evolution of prenatal care coverage in Brazil. In the year of 1995, more than 10% of Brazilian pregnant women did not have any prenatal care visit, and in 2015, only 2.2%. Less than half of the pregnant women used to attend seven or more prenatal care visits in 1995, increasing to 66.5% in 2015, showing an expansion of this coverage and the importance of the Brazilian Unified Health System (SUS), created in 1990, to the dissemination of this benefit^4^.

In 2013, the Ministry of Health (MH), the National Council of State Health Secretaries (CONASS), and the National Council of Municipal Health Secretaries (CONASEMS) agreed upon some health care indicators to strengthen the Integrated Planning of SUS and the implementation of the Public Health Action Organizational Contract (COAP)^5^.

The coverage of prenatal care is among these health care indicators, and we evaluated it here according to the five geographic macro regions, within SUS, using the data from the Birth in Brazil survey. We also analyzed the effects of inadequate prenatal care on the health of women and newborns.

## METHODS

This study is part of Birth in Brazil study, a research conducted in public and private services between 2011 and 2012.

Birth in Brazil is a nationwide hospital survey, with sampling in three stages of selection. In the first stage, hospitals with 500 or more deliveries per year were selected, stratified according to the macro regions of the country, by location (capital or non-capital) and by the type of hospital (public, private or mixed). In the second stage, the number of days necessary to perform 90 interviews with puerperal women in each hospital was defined. In the third and last stage, eligible puerperal women were selected. More information about the sample design is detailed by Vasconcellos et al.^6^

In total, Birth in Brazil included 23,894 puerperal women admitted for birth in the 266 selected hospitals and their newborns with any weight and gestational age, or stillbirths with weight ≥ 500g and/or gestational age ≥ 22 weeks of gestation.

For the current analysis, we included only 19,117 women with public-funded births, representing 80% of the national sample. Information were obtained from face-to-face interviews with women after birth, from medical records and from hand-held prenatal notes. Detailed information on data collection is available in another publication^7^.

Post-hoc calculations showed that—with a significance level of 5% and spontaneous preterm birth of 6% in the group of unexposed—the subgroup with the lowest sample size in the multiple regression (3,807 women with clinical or gestational complications) would have 90% power to detect an increased risk corresponding to an odds ratio (OR) ≥ 1.3. For the rarest outcomes, Apgar score in the 5th minute < 8 and maternal near miss, the smallest subgroup of women would have 90% power to detect increased risks corresponding to OR ≥ 2.0 and 2.5, respectively.

Prenatal care and access to maternity hospitals indicators were: type of prenatal care unit; trimester of onset of prenatal care; number of prenatal care appointments; had hand-held prenatal notes; had hand-held prenatal notes upon admission for birth; results of routine tests recorded in hand-held prenatal notes [fasting blood sugar test, urine (abnormal sediment elements—ASE), (VDRL), HIV and ultrasound]; received guidance on reference maternity for birth; birthed in the referenced maternity; pilgrimage for birth; type of hospital and location of hospital.

For the assessment of the prenatal care adequacy, three indicators were used. The first indicator considered the gestational trimester at the time of prenatal onset and the total number of appointments, adjusted for the gestational age at the birth. The onset of prenatal care was considered appropriate when performed until the 12th gestational week, according to the recommendation of the MH. The minimum calendar of the MH was used to calculate the adequacy of the number of appointments. The Ministry of Health recommends at least one appointment in the first gestational trimester, two appointments in the second and three appointments in the last trimester^2^. The number of appointments was considered adequate when the pregnant woman attended 100% of the minimum appointments planned for the gestational age at birth. The indicator was considered adequate when both early onset and number of prenatal appointments were adequate.

The second indicator, named overall adequacy 1, was created by Domingues et al.^8^, and it considers early onset, minimum number of appointments, routine examinations performed and guidance on reference maternity for birth. Prenatal care is considered adequate when the onset occurs until the 12th gestational week; the number of appointments is adequate (≥ 100% of the appointments planned for gestational age in birth); at least one test result must be recorded: fasting blood sugar, AES, VDRL, HIV and ultrasound; and there is guidance on the maternity reference for birth. The third indicator, named overall adequacy 2, considered the items of overall adequacy 1 plus having birthed in the hospital for which the woman was referred to during prenatal care.

The following outcomes were assessed: spontaneous preterm birth, provider-initiated preterm birth, low birth weight (LBW < 2,500g), intrauterine growth restriction (IUGR), Apgar in the 5th min < 8, neonatal near miss and maternal near miss. We classified as spontaneous preterm birth the ones with less than 37 gestational weeks in which the beginning of labor (L) was spontaneous or with premature rupture of the membranes. Provider-initiated preterm births were initiated either by induction or cesarean section before L. The women with rupture of membranes that gave birth by induced L or cesarean section before L were classified in the category spontaneous births. Labor was considered as induced if women with intact membranes received medical intervention to initiate uterine contraction before the onset of spontaneous labor. Surgeries that occurred without spontaneous or induced labor were considered cesarean sections before L. We used the 10th percentile of weight for gestational age at birth according to the Intergrowth criterion^9^ to classify the IUGR. Gestational age at birth was calculated by an algorithm based on estimates of early ultrasounds^10^.

The variable neonatal near miss was built based on recommendations from Pileggi-Castro et al.^11^, using information from hospital records. The presence of any of the following features indicates the neonatal near miss. Pragmatic criteria: Apgar score in the 5th min < 7, birth weight < 1,750g and gestational age < 33 weeks. Management criteria: antibiotic use, continuous positive airway pressure (CPAP), exposure to phototherapy in the first 72 hours; use of vasoactive drug, anticonvulsant, surfactant, cardiac massage, presence of hypoglycemia, and orotracheal intubation. The neonatal near miss is defined as a morbid event that almost resulted in newborn death in the first 28 days of life^12^.

Maternal near miss is defined by the World Health Organization (WHO) as “a woman who nearly died but survived a complication that occurred during pregnancy, childbirth or within 42 days after termination of pregnancy^13^.” WHO proposes a classification using 25 criteria based on the presence of cardiac, respiratory, renal, hepatic, neurological, coagulation-related and uterine dysfunction, which represent a set of clinical, laboratory and management identifiers^14^. These criteria defined by WHO were adopted for the identification of maternal near miss cases using information from hospital records. The identified cases were independently reviewed by two specialists, who aimed to identify possible inconsistencies in the extraction and/or typing of data from the medical records.

The following covariates were assessed: age (12–19, 20–34, ≥ 35 years), skin color (white, black, brown), years of schooling (≤ 7, 8–10, 11–14, ≥ 15), economic class (D+E, C, A+B), marital status (lives with a partner, does not live with a partner), wage labor (yes, no), parity (0, 1–2, ≥ 3), and clinical or gestational complications (hypertensive disorders such as chronic hypertension, pre-eclampsia and HELLP syndrome; eclampsia; pre-existing diabetes; gestational diabetes; kidney, cardiac, heart or autoimmune diseases; placenta praevia, and placenta abruption). The classification of complications was validated by two obstetricians using information from medical records.

In statistical analysis, macroregional differences in sociodemographic characteristics and clinical or gestational complications were tested by the χ^2^ test, with statistical significance (p < 0.05). The same procedure was used to assess inequalities, stratified by women with and without clinical or gestational complications.

We tested whether pilgrimage for birth and inadequacy of prenatal care were associated with neonatal outcomes in the two subgroups of women for the whole country. We used multiple non-conditional logistic regressions adjusting them by the following variables: region, age, schooling and parity. The selection of adjustment variables was due to their association, with statistical significance (p < 0.05), both with exposure and with the outcomes studied (data not shown). For maternal near miss we also adjusted for type of birth. The results were expressed in adjusted OR and 95% confidence interval (95%CI).

Because this is a complex sample, the statistical analysis was thorough reviewed, including a data weighting process calculated by the reverse of each woman probability of inclusion in the sample, with a procedure of calibration in each selection stratum to correct the effect of the sampling strategy. The Statistical Package for the Social Sciences (SPSS) software, version 21, was used in the statistical analysis.

## RESULTS

Table 1 compares the characteristics of the 19,117 women with public-funded births according to the macroregions of the country. In the North and Northeast regions, the frequency of births among adolescents, women with low education and lower economic level was higher than in the Southeast. The variable “does not live with a partner” was more common in the Southeast region, whereas “wage labor” was more frequent in the South. The North region concentrated the highest proportion of women with three or more previous deliveries. Clinical and gestational complications were more frequent in the South and Southeast, except for chronic hypertension, which was more prevalent in the Northeast, and gestational hypertensive syndromes, more frequent in the Midwest.

**Table 1:** Sociodemographic characteristics and clinical and gestational complications in women with birth funded by the Brazilian Unified Health System according to macroregions of Brazil, 2011–2012.

	North (n = 2,143)	Northeast (n = 5,683)	Southeast (n = 7,838)	South (n = 2,244)	Midwest (n = 1,209)	Brazil (n = 19,117)	p-value^a^

	%	%	%	%	%	%	
**Age in years**							
12–19	28.1	24.9	19.8	20.4	24.4	22.6	< 0.001
20–34	66.4	66.5	71.3	68.9	68.5	68.8	
≥ 35	5.6	8.6	8.9	10.7	7.1	8.5	
**Skin color**							
White	12.1	17.2	33.3	59.3	25.5	28.7	< 0.001
Black	6.0	11.6	10.8	6.9	8.3	9.9	
Brown	81.9	71.2	55.9	33.8	66.2	61.4	
**Years of schooling**							
≤ 7	37.5	43.3	24.7	30.6	24.0	32.3	< 0.001
8–10	30.9	24.7	30.6	32.2	35.5	29.4	
11–14	29.0	29.8	41.7	33.2	37.2	35.5	
≥ 15	2.7	2.2	3.0	3.9	3.3	2.9	
**Economic class**							
D+E	39.4	48.4	18.4	12.0	20.7	29.0	< 0.001
C	51.2	45.5	63.3	60.9	63.9	56.4	
A+B	9.4	6.1	18.3	27.1	15.4	14.5	
**Marital status**							
Does not live with a partner	19.3	18.2	24.5	14.9	19.2	20.6	< 0.001
Lives with a partner		81.8	75.5	85.1	80.8	79.4	
**Wage labor**							
No	75.0	75.1	63.3	57.4	64.2	67.5	< 0.001
Yes	25.0	24.9	36.7	42.6	35.8	32.5	
**Previous births**							
0	40.1	47.6	44.7	42.7	43.9	44.8	< 0.001
1–2	41.4	39.7	44.6	44.8	44.7	42.8	
≥ 3	18.5	12.6	10.7	12.5	11.4	12.4	
**Clinical or gestational complications**							
Pre-existing hypertension	1.5	2.8	3.1	2.8	1.6	2.7	< 0.001
Hypertensive syndromes^b^	8.2	10.5	11.6	11.5	11.7	10.9	< 0.001
Pre-existing diabetes	0.5	1.0	1.3	1.4	0.8	1.1	0.015
Gestational diabetes	7.6	5.5	9.5	11.1	5.5	8.0	< 0.001
Other chronic diseases^c^	0.6	0.8	0.8	0.8	1.2	0.8	0.465
Placenta praevia	0.7	0.4	0.4	0.8	0.4	0.4	0.261
Placental abruption	1.1	1.2	1.7	1.7	1.2	1.5	0.046

^a^ χ^2^ test.

^b^ Pre-eclampsia, HELLP syndrome, eclampsia.

^c^ Kidney, cardiac or autoimmune diseases.

Prenatal care showed important regional variations. Despite the high coverage, the proportion of women without any prenatal care was 60% higher in the North than the national average. The Southeast, South and Midwest regions had the highest prevalence of women with early prenatal onset, and the Southeast had the highest coverage of women with at least six prenatal appointments ( Table 2 ).

**Table 2:** Prenatal care of women with birth funded by the Brazilian Unified Health System according to maternal risk and macroregions in Brazil, 2011–2012.

	Women without complications							Women with complications							All women						

	N n = 1,773	NE n = 4,674	SE n = 6,143	S n = 1,727	MW n = 993	Brazil n = 15,310	p-value^a^	N n = 370	NE n = 1,008	SE n = 1,696	S n = 518	MW n = 215	Brazil n = 3,807	p-value^a^	N n = 2,143	NE n = 5,682	SE n = 7,839	S n = 2,245	MW n = 1,208	Brazil n = 19,117	p-value^a^

	%	%	%	%	%	%		%	%	%	%	%	%		%	%	%	%	%	%	
**Received prenatal care**																					
No	2.8	1.7	1.5	0.9	1.6	1.6	0.001	0.5	1.8	0.5	0.0	1.6	0.8	< 0.001	2.5	1.7	1.3	0.7	1.6	1.5	< 0.001
Yes	97.2	98.3	98.5	99.1	98.4	98.4		99.5	98.2	99.5	100.0	98.4	99.2		97.5	98.3	98.7	99.3	98.4	98.5	
**Type of basic unit** ^b^																					
FHP/birthing center/BHU	94.1	88.2	92.1	91.6	92.2	91.1	< 0.001	89.8	82.9	84.5	83.1	85.2	84.5	0.039	93.4	87.3	90.4	89.6	91.0	89.7	< 0.001
Outpatient clinic	5.9	11.8	7.9	8.4	7.8	8.9		11.2	17.1	15.5	16.9	14.8	15.5		6.6	12.7	9.6	11.4	9.0	10.3	
**Onset of prenatal care** ^b^																					
Up to12 weeks	45.2	52.0	59.3	57.1	65.2	55.6	< 0.001	49.7	57.7	63.7	59.7	65.7	60.3	< 0.001	46.0	52.2	60.1	57.6	65.0	56.5	< 0.001
Second trimester	49.8	43.9	36.8	37.5	31.4	40.2		46.2	38.7	32.0	37.0	32.9	35.9		49.2	43.0	35.8	37.5	31.4	39.3	
Third trimester	5.0	4.1	3.9	5.4	3.4	4.2		4.1	3.6	4.3	3.3	1.4	3.8		4.8	4.8	4.1	4.9	3.6	4.2	
**No. of prenatal appointments** ^b^																					
Between 1 and 3	17.7	14.1	9.0	9.9	8.5	11.7	< 0.001	15.1	8.4	5.7	6.9	4.3	7.4	< 0.001	17.2	13.1	8.3	9.2	7.7	10.9	< 0.001
Between 4 and 5	28.1	27.0	14.0	17.3	21.2	20.4		24.6	23.0	16.0	15.4	15.9	18.6		27.5	26.3	14.4	16.9	20.3	20.0	
6 or more	54.2	58.9		77.0	72.8	70.3	67.9		60.3	68.6		78.3	77.7	79.8	74.0	55.3	60.6	77.3	73.9	72.0	69.1
**Have received hand-held prenatal notes** ^b^																					
No	0.7	1.1	1.4	0.8	2.4	1.2	0.001	0.5	1.6	0.5	0.6	0.9	0.9	0.043	0.7	1.2	1.1	0.8	2.1	1.1	0.002
Yes	99.3	98.9	98.6	99.2	97.6	98.8		99.5	98.4	99.5	99.4	99.1	99.1		99.3	98.8	98.9	99.2	97.9	98.9	
**Took the hand-held prenatal notes on admission for birth** ^b^																					
No	35.9	25.8	20.0	16.4	54.0	25.4	< 0.001	22.9	17.9	13.5	10.4	38.4	16.5	< 0.001	33.6	24.4	18.5	15.0	51.2	23.6	< 0.001
Yes	64.1	74.2	80.0	83.6	46.0	74.6		77.1	82.1	86.5	89.6	61.6	83.5		66.4	75.6	81.5	85.0	48.8	76.4	
**Blood sugar level** ^c^																					
No	23.3	23.7	17.7	10.4	18.8	19.2	< 0.001	12.4	16.0	12.0	6.5	11.5	12.2	< 0.001	21.1	22.2	16.4	9.5	17.1	17.7	< 0.001
Yes, once	49.3	54.4	45.7	40.0	52.7	48.2		48.6	49.1	40.4	33.5	43.1	42.5		49.2	53.4	44.4	38.4	50.5	47.0	
Yes, twice	27.4	21.9	36.6	49.5	28.6	32.6		39.0	34.9	47.7	60.0	45.4	45.3		29.7	24.4	39.2	52.1	32.4	35.4	
**Urine (AES)** ^c^																					
No	24.8	19.1	14.9	8.4	18.3	16.4	< 0.001	12.5	13.7	11.5	5.8	10.0	11.2	< 0.001	22.3	18.0	14.1	7.8	16.4	15.3	< 0.001
Yes	75.2	80.9	85.1	91.6	81.7	83.6		87.5	86.3	88.5	94.2	90.0	88.8		77.7	82.0	85.9	92.2	83.6	84.7	
**Ultrasound** ^c^																					
No	32.9	18.9	9.6	9.2	14.3	14.8	< 0.001	27.0	15.8	8.2	6.7	8.5	11.6	< 0.001	31.7	18.3	9.3	8.6	13.0	14.1	< 0.001
Yes	67.1	81.1	90.4	90.8	85.7	85.2		73.0	84.2	91.8	93.3	91.5	88.4		68.3	81.7	90.7	91.4	87.0	85.9	
**VDRL test results during the pregnancy** ^c^																					
No	22.8	17.7	9.5	5.5	17.2	13.1	< 0.001	14.6	11.5	7.4	4.5	9.3	8.7	< 0.001	21.1	16.5	9.3	5.3	15.4	12.1	< 0.001
Yes, one	50.0	54.2	46.9	38.7	45.8	48.3		53.7	50.5	44.0	34.2	45.0	45.2		50.7	53.2	46.0	37.6	45.6	47.7	
Yes, two	27.2	28.1	43.6	55.8	37.0	38.6		31.7	38.0	48.6	61.3	45.7	46.1		28.2	30.3	44.7	57.1	39.0	40.2	
**HIV test results during the pregnancy** ^c^																					
No	31.6	35.5	14.3	8.1	19.6	21.9	< 0.001	28.8	28.4	11.5	6.9	15.4	16.8	< 0.001	31.0	34.1	13.7	7.8	18.6	20.7	< 0.001
Yes, one	50.5	51.3	53.4	49.8	47.9	51.8		52.8	51.5	55.4	48.0	48.4	52.8		51.0	51.4	53.8	49.4	48.1	52.1	
Yes, two	17.9	13.2	32.3	42.1	32.5	26.3		18.4	20.1	33.1	45.1	36.2	30.4		18.0	14.5	32.5	42.8	33.3	27.2	
**Bond with maternity hospital** ^b^																					
No	52.1	50.4	40.1	41.2	43.5	45.0	< 0.001	50.7	40.7	36.0	34.9	34.4	38.4	< 0.001	51.8	48.7	39.2	39.7	41.8	43.6	< 0.001
Yes	47.9	49.6	59.9	58.8	56.5	55.0		49.3	59.3	64.0	65.1	65.6	61.6		48.2	51.3	60.8	60.3	58.2	56.4	
**Birthed in the bonded maternity hospital** ^d^																					
No	14.2	23.3	19.7	8.7	16.0	18.5	< 0.001	14.5	25.0	17.9	10.4	19.0	18.4	< 0.001	14.2	23.7	19.3	9.0	16.6	18.5	< 0.001
Yes	85.8	76.7	80.3	91.3	84.0	81.5		85.5	75.0	82.1	89.6	81.0	81.6		85.8	76.3	80.7	91.0	83.4	81.5	
**Pilgrimage for birth**																					
No	80.0	67.2	78.5	89.9	76.4	76.4	< 0.001	78.2	65.0	81.0	87.0	75.8	77.0	< 0.001	79.7	66.9	79.1	89.2	76.3	76.5	< 0.001
Yes, one hospital	17.3	27.7	17.2	9.2	20.1	19.7		19.6	29.6	16.0	12.2	21.9	19.8		17.7	28.0	16.9	9.9	20.4	19.7	
Yes, two hospitals	2.7	5.1	4.3	0.9	3.5	3.9		2.2	5.4	3.0	0.8	2.3	3.2		2.6	5.1	4.0	0.9	3.3	3.8	
**Type of hospital**																					
Public	71.8	58.6	42.8	28.9	56.5	50.3	< 0.001	83.0	67.1	48.1	35.6	63.2	55.7	< 0.001	73.5	60.1	43.9	30.4	57.5	51.3	< 0.001
Mixed	28.2	41.4	57.2	71.1	43.5	49.7		17.0	32.9	51.9	64.4	36.8	44.3		26.5	39.9	56.1	69.6	42.5	48.7	
**Location of hospital**																					
Capital	45.2	34.7	32.2	22.3	64.3	35.4	< 0.001	45.8	50.2	39.2	30.5	70.4	43.4	< 0.001	45.2	37.5	33.7	24.1	65.2	37.0	< 0.001
Non-capital	54.8	65.3	67.8	77.7	35.7	64.6		54.2	49.8	60.8	69.5	29.6	56.6		54.8	62.5	66.3	75.9	34.8	63.0	

FHP: Family Health Program; BHU: basic health unit

^a^ χ^2^ test.

^b^ The women who attended the prenatal care are included in this analysis.

^c^ The women who attended the prenatal care and presented the prenatal card in the labor admission are included in this analysis.

^d^ The women who attended the prenatal care, presented the prenatal card in the labor admission and received guidance on the reference maternity for birth are included in this analysis .

The coverage of hand-held prenatal notes was almost universal. However, not all women took it to the hospital for admission for birth. The country coverage of at least one VDRL test and one HIV during pregnancy was 88% and 79%, respectively, with the North and Northeast regions with the lowest prevalence. The coverage of fasting blood sugar and ASE tests was near to 85%, with the South region with the highest prevalence. Considering ultrasound exams, the North region had the largest deficiency, with coverage under 70% ( Table 2 ).

Approximately half of the women were bonded with the maternity hospital during prenatal care. The South region stood out with more than 90% of women with births in the indicated maternities. Pilgrimage affected more than 20% of women in the country, reaching more than 30% in the Northeast ( Table 2 ).

Women with clinical or gestational complications had better prenatal indicators than women without complications. For the former, a higher prevalence of prenatal care, early onset and six or more appointments were observed. The coverage of VDRL and HIV tests was also higher for these women. In the Northeast, the difference observed among women with or without complications was more prominent than in other regions ( Table 2 ).

For the minimal adequacy of prenatal care, which considered both early onset and the minimum number of appointments, the Southeast and South regions had the highest prevalence. For the overall adequacy 1, the prevalence decreased substantially in all regions. When considering the most restricted criterion, the overall adequacy 2, the prevalence in the country was only 16%, with the Northeast region with the worst result, 10% ( Table 3 ). The Midwest region had the highest rate of cesarean section (49.5%), whereas the Northeast the lowest (41.6%). The Southeast had the lowest rate of antepartum cesarean section (6.8%), as observed in Table 3 .

**Table 3:** Adequacy of prenatal care and neonatal and maternal outcomes in women with birth funded by the Unified Health System according to maternal risk and macroregions of Brazil, 2011–2012.

	Women without complications							Women with complications							All women						

	N n = 1,773	NE n = 4,674	SE n = 6,143	S n = 1,727	MW n = 993	Brazil n = 15,310	p-value^a^	N n = 1,773	NE n = 4,674	SE n = 6,143	S n = 1,727	MW n = 993	Brazil n = 15,310	p-value^a^	N n = 1,773	NE n = 4,674	SE n = 6,143	S n = 1,727	MW n = 993	Brazil n = 15,310	p-value^a^
		
	%	%	%	%	%	%		%	%	%	%	%	%		%	%	%	%	%	%	
**Adequacy of prenatal care** ^b^	47.3	52.6	67.1	65.8	63.4	60.1	< 0.001	55.5	64.5	72.0	69.5	72.6	68.2	< 0.001	48.7	54.7	68.2	66.7	65.0	61.7	< 0.001
**Adequacy of overall prenatal care 1** ^c^	10.1	11.4	21.6	24.6	18.5	17.7	< 0.001	12.0	21.0	29.4	29.4	26.9	25.6	< 0.001	10.5	13.3	23.4	25.7	20.4	19.4	< 0.001
**Adequacy of overall prenatal care 2** ^d^	9.6	8.8	17.3	22.5	15.1	14.5	< 0.001	11.7	16.2	24.0	26.1	24.8	21.2	< 0.001	10.0	10.2	18.9	23.4	17.3	16.0	< 0.001
**Type of birth**																					
Vaginal	59.1	63.0	62.0	59.8	57.2	61.4	< 0.001	36.2	37.2	38.8	45.3	20.6	37.9	< 0.001	55.1	58.4	57.0	56.5	50.5	56.7	< 0.001
Intrapartum cesarean section	31.7	27.2	31.4	32.0	33.7	30.4		52.6	50.6	53.4	45.3	68.2	52.4		35.3	31.4	36.2	35.1	40.0	34.8	
Antepartum cesarean section	9.3	9.8	6.6	8.2	9.1	8.2		11.1	12.2	7.8	9.4	11.2	9.7		9.6	10.2	6.8	8.5	9.5	8.5	
																					
**Outcomes**																					
Spontaneous preterm birth	8.5	9.0	6.7	7.4	8.4	7.8	< 0.001	9.8	8.1	5.8	8.4	5.8	7.2	0.023	8.8	8.9	6.5	7.6	7.9	7.7	< 0.001
Provided-initiated preterm birth	3.5	1.9	2.1	2.5	1.4	2.2	< 0.001	11.9	11.8	11.9	10.5	12.1	11.7	0.949	4.9	3.7	4.2	4.4	3.4	4.1	0.096
Low birth weight (< 2,500g)	7.9	8.9	7.6	7.7	7.8	8.1	0.124	17.9	18.7	13.5	12.7	17.6	15.4	0.001	9.6	10.7	8.9	8.9	9.6	9.5	0.001
IUGR (< 10th percentile)	5.9	7.7	7.9	7.0	8.8	7.6	< 0.001	11.6	10.3	7.1	6.3	8.1	8.4	< 0.001	6.9	8.2	7.8	6.8	8.7	7.7	< 0.001
Apgar in the 5th minute < 8	1.6	3.4	1.9	1.3	1.2	2.2	< 0.001	3.2	3.0	2.5	1.8	2.9	2.6	0.615	1.9	3.3	2.0	1.4	1.5	2.3	< 0.001
Neonatal near miss	9.2	9.1	10.4	10.5	8.9	9.8	0.100	15.9	17.6	18.9	17.2	17.9	18.0	0.670	10.3	10.6	12.2	12.0	10.6	11.4	0.061
Maternal near miss	0.5	0.3	0.3	0.6	0.2	0.4	0.318	3.4	5.5	3.3	3.3	7.6	4.1	0.004	1.0	1.2	1.0	1.2	1.6	1.1	0.333

IUGR: intrauterine growth restriction (< 10th percentile in intergrowth curve)

^a^ χ^2^ test.

^b^ The women who attended the prenatal care are included in this analysis. Prenatal care was considered adequate when it started until the 12th gestational week and had an adequate number (100%) of appointments for gestational age at birth, considering the schedule of six appointments.

^c^ The women who attended the prenatal care and took the hand-held prenatal notes for admission for birth are included in this analysis. Prenatal care was considered adequate when it started until the 12th gestational week and had an adequate number (100%) of appointments for gestational age at birth, considering the schedule of six appointments, there was at least one result of each recommended tests in the prenatal routine and women received guidance on the reference maternity for birth care.

^d^ The women who attended the prenatal care, took the hand-held prenatal notes for admission for birth and received guidance on the reference maternity for birth are included in this analysis .

Prenatal care was considered adequate when it started until the 12th gestational week and had an adequate number (100%) of appointments for gestational age at birth, considering the schedule of six appointments, there was at least one result of each recommended tests in the prenatal routine, women received guidance on the reference maternity for birth care, and birth occurred in the reference maternity.

The North, Northeast and Midwest regions had the worst neonatal results, mainly for spontaneous preterm birth, LBW and IUGR. We found more regional inequalities and worse outcomes among women with complications ( Table 3 ).

Pilgrimage was associated with all neonatal outcomes, except IUGR, even after adjustment for confounding variables. The odds ratios ranged from 1.48 (95%CI 1.23–1.78) for neonatal near miss to 1.62 (95%CI 1.27–2.06) for provider-initiated preterm birth. Among women with clinical or gestational complications, pilgrimage was strongly associated with provider-initiated preterm birth and Apgar in the 5th min < 8, with OR of 1.98 (95%CI 1.49–2.65) and 2.19 (95%CI 1.31–3.68), respectively. The prenatal inadequacy was associated with spontaneous preterm birth in both groups of women ( Table 4 ).

**Table 4:** Neonatal and maternal outcomes associated with journey and inadequate prenatal care in women with birth financed by the Brazilian Unified Health System according to maternal risk in Brazil. 2011–2012.

	Spontaneous preterm birth	Provided-initiated preterm birth	LBW	IUGR	Apgar 5 min < 8	Neonatal near miss	Maternal near miss
						
	OR^a^ (95%CI)	OR^a^ (95%CI)	OR^a^ (95%CI)	OR^a^ (95%CI)	OR^a^ (95%CI)	OR^a^ (95%CI)	OR^a^ (95%CI)
**Pilgrimage for birth**							
All women	1.61 (1.29–2.01)	1.62 (1.27–2.06)	1.56 (1.28–1.89)	1.19 (0.95–1.49)	1.56 (1.19–2.03)	1.48 (1.23–1.78)	1.38 (0.81–2.36)
Women without complications	1.60 (1.25–2.04)	1.32 (0.92–1.91)	1.52 (1.23–1.87)	1.14 (0.86–1.51)	1.41 (1.03–1.94)	1.50 (1.21–1.85)	1.34 (0.70–2.56)
Women with complications	1.67 (1.06–2.61)	1.98 (1.49–2.65)	1.69 (1.23–2.31)	1.37 (1.03–1.83)	2.19 (1.31–3.68)	1.45 (1.16–1.81)	1.39 (0.70–2.77)
**Inadequate prenatal care (onset and number of appointments)**							
All women	1.51 (1.24–1.83)	1.06 (0.73–1.55)	1.25 (0.99–1.56)	1.11 (0.88–1.38)	0.96 (0.68–1.36)	1.14 (0.95–1.37)	0.96 (0.54–1.68)
Women without complications	1.47 (1.18–1.82)	0.95 (0.62–1.46)	1.21 (0.94–1.54)	1.19 (0.95–1.49)	1.00 (0.69–1.43)	1.08 (0.88–1.34)	0.82 (0.29–2.36)
Women with complications	1.73 (1.02–2.94)	1.14 (0.66–1.97)	1.32 (0.90–1.92)	1.14 (0.86–1.51)	0.81 (0.39–1.71)	1.28 (0.85–1.93)	0.97 (0.55–1.73)

OR: odds ratio; 95%CI: 95% confidence interval; LBW: low birth weight (< 2,500g); IUGR: intrauterine growth restriction (< 10th percentile in the Intergrowth curve).

^a^ Adjusted by region, age, schooling and parity.

^a^ Adjusted by region, age, schooling, parity and type of birth.

## DISCUSSION

Prenatal care coverage in Brazil is almost universal for women using SUS, considering at least one prenatal appointment. However, this panorama changes as other parameters are progressively included. By including recommendations such as a minimum set of exams and the bond with the maternity hospital for birth, prenatal adequacy is reduced to slightly over a quarter of women, being further reduced if we consider effective bonding with the maternity hospital for birth.

Tomasi et al.^15^ analyzed prenatal care in SUS in the same period of this study, observing that 89% of the pregnant women had six or more appointments, but only 15% received adequate prenatal care. The differences in coverage percentages between the two studies derive from two main reasons. Firstly, Tomasi’s study included only women aged 18 years or older in basic health units (BHU) by occasion of their evaluation; whereas Birth in Brazil included a representative sample of women at the time of birth. Secondly, the criterion used for prenatal adequacy was different in both studies. Since 99% of the deliveries occurred in hospitals, our data are closer to the Brazilian prenatal coverage.

Our results indicate that the continuity and quality of care provided by SUS is still deficient. In the state of São Paulo, Monteiro et al.^16^ verified that, despite a nearly universal access to health care services, there were still problems related to quality, which were experienced mainly by lower socioeconomic groups and users of SUS.

The social and economic inequalities among the geographical regions of the country are evident in this study. In the sphere of reproductive health, women are younger in the most disadvantaged regions, with a higher proportion of teenage pregnancy and greater parity. Women from the South and Southeast regions had higher proportions of clinical complications, probably because they were older and had more access to clinical diagnosis. However, in the North region, the proportion of women with no prenatal care was the same for both groups of women, with and without obstetric complications. This poorer performance in the North region may be due to geographical difficulties, large distances and barriers in the access of centers for diagnosis and treatment, absence of qualified professionals, etc. The North region had the highest proportion of home births, that when performed by unqualified professionals, associate with higher infant mortality rates^17^. Regarding prenatal care in Brazil, Viellas et al.^18^ found that the low coverage and late onset of prenatal care in women with low schooling from the North and Northeast regions were more related to barriers in the access to care than with the lack of knowledge of pregnancy or personal problems.

In this study, pregnant women with inadequate prenatal care were more susceptible to give birth to preterm newborns spontaneously. A study with this same sample of women observed that spontaneous preterm birth was associated with poverty and inadequacy of prenatal care^19^, factors which contribute to the maintenance of the high infant mortality rates in the country^20^, since preterm birth is the greatest risk factor for morbidity and mortality in the first year of life^21^.

Furthermore, our study identified the lack of bond between the levels of outpatient and hospital care, an important aspect, since knowing the maternity hospital for birth contributes to the well-being of women and to the progress of the labor^22^. The law of maternity bond has completed ten years, but it has not been properly implemented in the country. Notably, the Southern region has achieved effective bond for more than 90% of pregnant women, thus, this region evidences a better organization of the system, better care coordination, better continuity and hierarchization of actions in maternal and child health.

The cesarean section rate in all regions was high — especially for women with obstetric complications —, 75% higher than in the low-risk group. The average rate of 30.4% in the low-risk group approaches the value of the U.S. overall rate and is above the rate of European countries, whose rate is between 20% and 25%^23^. The Southeast region, which had the lowest rate of intrapartum cesarean section, also had the lowest overall cesarean section rate in the public sector of SUS (data not shown). In European countries, intrapartum cesarean sections are higher than antepartum, differently from what occurs in Brazil, where antepartum or elective cesarean section predominates^24^. The findings from the Southeast region may be a consequence of the ongoing movement of change in the birth care model of their SUS hospitals; focused on the best scientific evidence, without disregarding the women’s movement for a less medicalized birth care.

Women who had clinical or gestational complications received better prenatal care than low-risk ones. This fact shows that primary care has been effective in identifying these problems and in attending at-risk pregnant women. However, the system has failed the process of integrality care, not giving continuity to access to maternity care.

The failure of the system in the coordination and integrality of care at the time of birth, with many women in pilgrimage, associated with great damage to newborns. In obstetrics, the role of delays in care is known. In the 1990s, English authors proposed a theoretical model called “three delays” that classifies the delays in: phase I, the delay in deciding to seek care; phase II, the delay in reaching an appropriate care unit; and phase III, the delay in receiving adequate care at the reference institution^25^. When a woman search for more than one health service to give birth, we are certainly facing phase II and III. Pacagnella et al.^26^ have used these concepts and analyzed the role of delays in maternal morbidity in Brazil and found an association between this outcome and the delays in care. In the current analysis, pilgrimage associated with nearly all negative outcomes in the newborn, especially in the group of pregnant women with complications, possibly because they require more clinical interventions.

A recent analysis with data from Birth in Brazil showed that in SUS 32% of women with a obstetric risk were treated in hospitals without intensive care units, while 29.5% of low-risk patients delivered in hospitals with this type of resource^27^. This fact shows that the system is not adequately articulated to provide high complexity attention when necessary, although the system offers unnecessary support to those who do not need it. The consequences of this disarticulation can be seen in this study and deserve attention from managers to avoid suffering, complications and abbreviation of lives.

Prenatal care is a programmatic action typical of primary care, and the results of this study prove this fact and its relationship with obstetric results. Since 90% of the interviewees did their prenatal care in the basic health network, the qualification actions of the teams and work processes play a fundamental role in improving care for the baby and pregnant women. Fachini et al.^28^ highlight the importance of increasing the effectiveness of the Family Health Strategy, considering its mediating effect on health care.

In conclusion, regional inequalities, barriers to access and inadequate prenatal care persist, thus, they contribute to adverse outcomes for newborns. The improvement in the quality of prenatal care and the coordination and integrality of care at the time of birth have a potential impact on preterm birth rates and consequently on the reduction of infant morbidity and mortality rates in the country.
