# Supplementary material for: Prenatal care in the Brazilian public health services
Source: Rev Saude Publica. 2020 Jan 14;54:8. doi: 10.11606/s1518-8787.2020054001458 (PMC6961968; doi:10.11606/s1518-8787.2020054001458)
Supplement: Translation

# Translation: Assistência pré-natal na rede pública do Brasil

**Authors:** Maria do Carmo Leal, Ana Paula Esteves-Pereira, Elaine Fernandes Viellas, Rosa Maria Soares Madeira Domingues, Silvana Granado Nogueira da Gama

**Affiliations:** I Fundação Oswaldo Cruz. Escola Nacional de Saúde Pública (ENSP/Fiocruz). Departamento de Epidemiologia e Métodos Quantitativos em Saúde (DEMQS). Rio de Janeiro, RJ, Brasil.; II Fundação Oswaldo Cruz. Instituto Nacional de Infectologia Evandro Chagas. Laboratório de Pesquisa Clínica em HIV/Aids (INI/Fiocruz). Rio de Janeiro, RJ, Brasil.

**Keywords:** Cuidado Pré-Natal, Serviços de Saúde Materno-Infantil, Qualidade, Acesso e Avaliação da Assistência à Saúde, Fatores Socioeconômicos, Disparidades nos Níveis de Saúde

## Abstract

**OBJETIVO:**

Verificar desigualdades regionais no acesso e na qualidade da atenção ao pré-natal e ao parto nos serviços públicos de saúde no Brasil e a sua associação com a saúde perinatal.

**MÉTODOS:**

Nascer no Brasil foi uma pesquisa nacional de base hospitalar realizada entre 2011 e 2012, que incluiu 19.117 mulheres com pagamento público do parto. Diferenças regionais nas características sociodemográficas e obstétricas, bem como as diferenças no acesso e qualidade do pré-natal e parto foram testadas pelo teste do χ^2^. Foram avaliados os desfechos: prematuridade espontânea, prematuridade iniciada por intervenção obstétrica, baixo peso ao nascer, crescimento intrauterino restrito, Apgar no 5º min < 8, *near miss* neonatal e *near miss* materno. Para a análise dos desfechos perinatais associados, foram utilizadas regressões logísticas múltiplas e não condicionais, com resultados expressos em *odds ratio* ajustada e intervalo de confiança de 95%.

**RESULTADOS:**

As desigualdades regionais ainda são evidentes no Brasil, no que diz respeito ao acesso e qualidade do atendimento pré-natal e ao parto entre as usuárias dos serviços públicos. A peregrinação para o parto se associou a todos os desfechos perinatais estudados, exceto para crescimento intrauterino restrito. As *odds ratios* variaram de 1,48 (IC95% 1,23–1,78) para *near miss* neonatal a 1,62 (IC95% 1,27–2,06) para prematuridade iniciada por intervenção obstétrica. Entre as mulheres com alguma complicação clínica ou obstétrica, a peregrinação se associou ainda mais com a prematuridade iniciada por intervenção e com Apgar no 5º min < 8, *odds ratio* de 1,98 (IC95% 1,49–2,65) e 2,19 (IC95% 1,31–3,68), respectivamente. A inadequação do pré-natal se associou à prematuridade espontânea em ambos os grupos de mulheres

**CONCLUSÃO:**

Melhorar a qualidade do pré-natal, a coordenação e a integralidade do atendimento no momento do parto têm um impacto potencial nas taxas de prematuridade e, consequentemente, na redução das taxas de morbimortalidade infantil no país.

## INTRODUÇÃO

A atenção pré-natal se constitui em um conjunto de ações que são simultaneamente preventivas, promotoras de saúde, diagnósticas e curativas, visando o bom desfecho da gestação para a mulher e seu(s) filho(s)^1^.

A recomendação brasileira, no ano de 2012, era de no mínimo seis consultas de pré-natal, com uso de vacinas, realização de testes de diagnóstico laboratorial de exames de rotina, oferta de suplementos e tratamento medicamentoso para os problemas encontrados^2^, com todos os procedimentos registrados na Caderneta da Gestante, importante para a referência e a contrarreferência no momento do parto. A vinculação da gestante ao local do parto também é uma recomendação, para prevenir a peregrinação por busca de atenção hospitalar durante o trabalho de parto^3^.

Os dados do Sistema de Informações sobre Nascidos Vivos (Sinasc) mostram a evolução da cobertura da atenção pré-natal no Brasil, de nenhuma consulta para mais de 10% das gestantes brasileiras há vinte anos, em 1995, caindo para 2,2% em 2015. Menos da metade das mulheres grávidas faziam sete ou mais consultas, e esse percentual aumentou para 66,5% em 2015, mostrando a expansão dessa cobertura e a importância do Sistema Único de Saúde (SUS), criado em 1990, na difusão desse benefício^4^.

O Ministério da Saúde (MS), o Conselho Nacional de Secretários de Saúde (Conass) e o Conselho Nacional de Secretários Municipais de Saúde (Conasems) pactuaram, em 2013, indicadores da atenção à saúde com vistas ao fortalecimento do Planejamento Integrado do SUS e à implementação do Contrato Organizativo de Ação Pública da Saúde (Coap)^5^.

Entre os indicadores pactuados, está a cobertura adequada da atenção pré-natal, que foi avaliada nesse artigo por grandes regiões geográficas, no SUS, com dados da pesquisa Nascer no Brasil. Também foram analisados os efeitos da inadequação da atenção pré-natal sobre a saúde da mulher e do recém-nascido.

## MÉTODOS

Este estudo é parte da pesquisa Nascer no Brasil, conduzida em serviços públicos e privados entre 2011 e 2012.

É uma pesquisa hospitalar, de âmbito nacional, com amostragem em três estágios de seleção. No primeiro estágio foram selecionados hospitais com 500 ou mais partos ao ano, estratificados segundo as macrorregiões do país, pela localização (capital ou não capital) e pelo tipo de hospital (público, privado ou misto). No segundo foi definido o número de dias necessários para realização de 90 entrevistas com puérperas em cada hospital. No terceiro e último estágio, a seleção das puérperas elegíveis. Mais informações sobre o desenho amostral são detalhadas por Vasconcellos et al.^6^

Participaram do estudo 23.894 puérperas admitidas por ocasião do parto nas 266 maternidades selecionadas e seus conceptos vivos com qualquer peso e idade gestacional, ou natimortos com peso ao nascer ≥ 500 g e/ou idade gestacional ≥ 22 semanas de gestação.

Cálculos post-hoc mostraram que, com um nível de significância de 5% e prematuridade espontânea de 6% nos não expostos, o subgrupo com menor tamanho amostral da regressão múltipla (3.807 mulheres com intercorrências clínicas ou gestacionais) teria 90% de poder para detectar um risco aumentado correspondente a um *odds ratio* (OR) ≥ 1,3. Para os desfechos mais raros, escore Apgar no 5º minuto < 8 e *near miss* materno, o menor subgrupo de mulheres teria 90% de poder para detectar riscos aumentados correspondentes a OR ≥ 2,0 e 2,5, respectivamente.

Para a presente análise, foram incluídas apenas as mulheres com parto pago pelo SUS, representando 80% da amostra nacional. Foram utilizadas informações obtidas por meio de entrevistas presenciais após o parto, dados do prontuário e dos cartões de pré-natal. Informações detalhadas sobre a coleta de dados estão disponíveis em outra publicação^7^.

Os indicadores da assistência pré-natal e acesso à maternidade foram: tipo de unidade do pré-natal, trimestre de início do pré-natal, número de consultas, recebimento do cartão da gestante, apresentação do cartão da gestante na admissão para o parto, resultado de exames de glicemia de jejum, urina (elementos anormais do sedimento – EAS), sorologia para sífilis (VDRL) e HIV, ultrassonografia, recebimento da orientação sobre a maternidade de referência para o parto, parto na maternidade referenciada, peregrinação para o parto, tipo e localidade do hospital do parto.

Para a avaliação da adequação da assistência pré-natal, utilizamos três indicadores. O primeiro levou em consideração o trimestre gestacional na época de início do pré-natal e o número total de consultas recebidas corrigido segundo a idade gestacional no momento do parto. Foi considerado adequado o início do acompanhamento pré-natal quando realizado até a 12ª semana gestacional, conforme recomendação do MS. Para cálculo da adequação do número de consultas, foi utilizado o calendário mínimo de consultas recomendado pelo MS, que preconiza a realização de pelo menos uma consulta no primeiro trimestre gestacional, duas no segundo e três no último^2^. O número de consultas foi considerado adequado quando a gestante realizou 100% das consultas mínimas previstas para a idade gestacional no momento do parto. O indicador foi considerado adequado quando ambos o início precoce e o número de consultas de pré-natal foram adequados.

O segundo indicador, denominado adequação global 1, foi elaborado por Domingues et al.^8^e considera o início precoce, número mínimo de consultas, exames de rotina realizados e orientação sobre a maternidade de referência para o parto. O pré-natal é considerado adequado quando o início ocorre até a 12ª semana gestacional; o número de consultas é adequado (≥ 100% das consultas previstas para a idade gestacional no parto); há registro de pelo menos um resultado de cada um dos exames: glicemia de jejum, EAS, VDRL, HIV e ultrassonografia; e há orientação sobre a maternidade de referência para o parto. O terceiro indicador, denominado adequação global 2, considerou os itens da adequação global 1 mais o recebimento de assistência ao parto no hospital para o qual a mulher foi referenciada durante do pré-natal.

Foram avaliados os desfechos: prematuridade espontânea, prematuridade iniciada por intervenção obstétrica, baixo peso ao nascer (BPN < 2.500 g), crescimento intrauterino restrito (CIUR), Apgar no 5º min < 8, *near miss* neonatal e *near miss* materno. Classificamos como prematuridade espontânea os nascimentos com menos de 37 semanas gestacionais em que o início do trabalho de parto (TP) foi espontâneo ou com ruptura prematura das membranas. Já os nascimentos prematuros por intervenção ou foram iniciados por indução ou pela cesariana antes do TP. Mulheres com ruptura de membranas que deram à luz através do TP induzido ou pela cesariana antes do TP foram classificadas sob a categoria nascimentos espontâneos. O TP foi considerado induzido em mulheres com membranas intactas e que receberam intervenção médica para iniciar a contração uterina antes do início do TP espontâneo. Foram consideradas cesáreas antes do TP as cirurgias que ocorreram sem TP espontâneo ou induzido. Para a classificação do CIUR, utilizamos o percentil 10 de peso segundo a idade gestacional no nascimento de acordo com o critério Intergrowth^9^. A idade gestacional no nascimento foi calculada por um algoritmo que baseou-se principalmente em estimativas de ultrassonografia precoce^10^.

A variável *near miss* neonatal foi construída baseada em recomendações de Pileggi-Castro et al.^11^, utilizando-se informações dos prontuários hospitalares. A presença de qualquer uma das características a seguir indica a presença de *near miss* neonatal. Critérios pragmáticos: escore de Apgar no 5º min < 7, peso ao nascer < 1.750 gramas e idade gestacional < 33 semanas. Critérios de manejo: uso de antibiótico, pressão positiva contínua nas vias aéreas (CPAP), fototerapia nas primeiras 72 horas, droga vasoativa, anticonvulsivante, surfactante, recebimento de massagem cardíaca, presença de hipoglicemia e intubação orotraqueal. Por definição, *near miss* neonatal representa um evento mórbido que quase resultou na morte do recém-nascido nos primeiros 28 dias de vida^12^.

*Near miss* materno é definido pela Organização Mundial da Saúde (OMS) como “uma mulher que quase morreu, mas sobreviveu à complicação que ocorreu durante a gravidez, parto ou até 42 dias após o fim da gestação”^13^. Propõe uma classificação utilizando 25 critérios baseados na presença de disfunção de cardíaca, respiratória, renal, hepática, neurológica, da coagulação e uterina, que representam um conjunto de identificadores clínicos, laboratoriais e de gerenciamento^14^. Para a identificação dos casos de *near miss* materno, foram adotados esses critérios definidos pela OMS, utilizando-se informações dos prontuários hospitalares. Os casos identificados foram revistos por dois especialistas de forma independente, visando identificar possíveis inconsistências na extração e/ou na digitação de dados do prontuário.

Como covariáveis foram avaliadas: idade (12–19, 20–34, ≥ 35 anos), cor da pele (branca, preta, parda), anos de estudo (≤ 7, 8–10, 11–14, ≥ 15), classe econômica (D+E, C, A+B), situação conjugal (mora, não mora com companheiro), trabalho remunerado (sim, não), paridade (0, 1–2, ≥ 3) e intercorrências clínicas ou gestacionais (distúrbios hipertensivos como hipertensão crônica, pré-eclâmpsia e síndrome HELLP; eclâmpsia; diabetes pré-existente; diabetes gestacional; doenças renais, cardíacas ou autoimunes; placenta prévia e descolamento prematuro de placenta). A classificação das intercorrências foi validada por dois obstetras usando informações do prontuário médico.

Na análise estatística, as diferenças macrorregionais nas características sociodemográficas e nas intercorrências clínicas ou gestacionais foram testadas pelo teste χ2, com significância estatística (p < 0,05). O mesmo procedimento foi utilizado para avaliar desigualdades, estratificando por mulheres com e sem intercorrências clínicas ou gestacionais.

Para o conjunto do país, testamos se a peregrinação e a inadequação do pré-natal se associavam aos desfechos neonatais nos dois subgrupos de mulheres. Para isso, utilizamos regressões logísticas múltiplas não condicionais e ajustamos pelas variáveis: região, idade, escolaridade e paridade. A seleção das variáveis de ajuste se deu pela associação delas, com significância estatística (p < 0,05), tanto com a exposição quanto com os desfechos estudados (dados não mostrados). Para o desfecho *near miss* materno, ajustamos também pelo tipo de parto. Os resultados foram expressos em OR ajustada e intervalo de confiança de 95% (IC95%).

Por tratar-se de uma amostra complexa, alguns cuidados foram levados em consideração em toda a análise estatística, que incluiu um processo de ponderação de dados calculado pelo inverso da probabilidade de inclusão de cada mulher na amostra, com um procedimento de calibração em cada estrato de seleção, a fim de corrigir o efeito do plano amostral. O software *Statistical Package for the Social Sciences* (SPSS), versão 21, foi utilizado na realização das análises estatísticas.

## RESULTADOS

Na Tabela 1 comparamos as características das 19.117 mulheres com parto pago pelo SUS segundo as macrorregiões do país. Nas regiões Norte e Nordeste, a frequência de partos entre adolescentes, mulheres de baixa escolaridade e de menor nível econômico foi maior que na região Sudeste. Não morar com companheiro foi mais comum na região Sudeste, enquanto o trabalho remunerado foi mais frequente no Sul. O Norte concentrou a maior proporção de mulheres com três ou mais partos anteriores. As intercorrências clínicas e gestacionais foram mais frequentes no Sul e Sudeste, exceto hipertensão crônica, que foi mais prevalente no Nordeste, e as síndromes hipertensivas gestacionais, mais frequentes no Centro-Oeste.

**Tabela 1:** Características sociodemográficas e intercorrências clínicas e gestacionais em mulheres com parto pago pelo Sistema Único de Saúde segundo macrorregiões do Brasil, 2011–2012.

	Norte (n = 2.143)	Nordeste (n = 5.683)	Sudeste (n = 7.838)	Sul (n = 2.244)	Centro-Oeste (n = 1.209)	Brasil (n = 19.117)	P-valor^a^
					
	%	%	%	%	%	%	
**Idade em anos**							
12–19	28,1	24,9	19,8	20,4	24,4	22,6	< 0,001
20–34	66,4	66,5	71,3	68,9	68,5	68,8	
≥ 35	5,6	8,6	8,9	10,7	7,1	8,5	
**Cor da pele**							
Branca	12,1	17,2	33,3	59,3	25,5	28,7	< 0,001
Preta	6,0	11,6	10,8	6,9	8,3	9,9	
Parda	81,9	71,2	55,9	33,8	66,2	61,4	
**Anos de escolaridade**							
≤ 7	37,5	43,3	24,7	30,6	24,0	32,3	< 0,001
8–10	30,9	24,7	30,6	32,2	35,5	29,4	
11–14	29,0	29,8	41,7	33,2	37,2	35,5	
≥ 15	2,7	2,2	3,0	3,9	3,3	2,9	
**Classe econômica**							
D+E	39,4	48,4	18,4	12,0	20,7	29,0	< 0,001
C	51,2	45,5	63,3	60,9	63,9	56,4	
A+B	9,4	6,1	18,3	27,1	15,4	14,5	
**Situação conjugal**							
Não mora com companheiro	19,3	18,2	24,5	14,9	19,2	20,6	< 0,001
Mora com companheiro	80,7	81,8	75,5	85,1	80,8	79,4	
**Trabalho remunerado**							
Não	75,0	75,1	63,3	57,4	64,2	67,5	< 0,001
Sim	25,0	24,9	36,7	42,6	35,8	32,5	
**Partos anteriores**							
0	40,1	47,6	44,7	42,7	43,9	44,8	< 0,001
1–2	41,4	39,7	44,6	44,8	44,7	42,8	
≥ 3	18,5	12,6	10,7	12,5	11,4	12,4	
**Intercorrências clínicas ou gestacionais**							
Hipertensão pré-existente	1,5	2,8	3,1	2,8	1,6	2,7	< 0,001
Síndromes hipertensivas^b^	8,2	10,5	11,6	11,5	11,7	10,9	< 0,001
Diabetes pré-existente	0,5	1,0	1,3	1,4	0,8	1,1	0,015
Diabetes gestacional	7,6	5,5	9,5	11,1	5,5	8,0	< 0,001
Outras doenças crônicas^c^	0,6	0,8	0,8	0,8	1,2	0,8	0,465
Placenta prévia	0,7	0,4	0,4	0,8	0,4	0,4	0,261
Descolamento prematuro da placenta	1,1	1,2	1,7	1,7	1,2	1,5	0,046

^a^Teste do χ^2^.

^b^Pré-eclâmpsia, síndrome HELLP, eclâmpsia.

^c^Doenças renais, cardíacas ou autoimunes.

A atenção pré-natal apresentou variações regionais importantes. Apesar da cobertura elevada, a proporção de mulheres sem nenhuma assistência pré-natal foi 60% maior no Norte que a média nacional. As regiões Sudeste, Sul e Centro-Oeste tiveram maior prevalência de mulheres com início precoce do pré-natal, e a Sudeste, a maior cobertura de mulheres com pelo menos seis consultas de pré-natal ( Tabela 2 ).

**Tabela 2:** Assistência pré-natal de mulheres com parto pago pelo Sistema Único de Saúde segundo risco materno e macrorregiões do Brasil, 2011–2012.

	Mulheres sem intercorrências							Mulheres com intercorrências							Todas as mulheres						
		
	N n = 1.773	NE n = 4.674	SE n = 6.143	S n = 1.727	CO n = 993	Brasil n = 15.310	P-valor^a^	N n = 370	NE n = 1.008	SE n = 1.696	S n = 518	CO n = 215	Brasil n = 3.807	P-valor^a^	N n = 2.143	NE n = 5.682	SE n = 7.839	S n = 2.245	CO n = 1.208	Brasil n = 19.117	P- valor^a^
			
	%	%	%	%	%	%		%	%	%	%	%	%		%	%	%	%	%	%	
**Teve assistência pré-natal**																					
Não	2,8	1,7	1,5	0,9	1,6	1,6	0,001	0,5	1,8	0,5	0,0	1,6	0,8	< 0,001	2,5	1,7	1,3	0,7	1,6	1,5	< 0,001
Sim	97,2	98,3	98,5	99,1	98,4	98,4		99,5	98,2	99,5	100,0	98,4	99,2		97,5	98,3	98,7	99,3	98,4	98,5	
**Tipo de unidade** ^b^																					
PSF/casa de parto/UBS	94,1	88,2	92,1	91,6	92,2	91,1	< 0,001	89,8	82,9	84,5	83,1	85,2	84,5	0,039	93,4	87,3	90,4	89,6	91,0	89,7	< 0,001
Ambulatório hospitalar	5,9	11,8	7,9	8,4	7,8	8,9		11,2	17,1	15,5	16,9	14,8	15,5		6,6	12,7	9,6	11,4	9,0	10,3	
**Início do pré-natal** ^b^																					
Até 12 semanas	45,2	52,0	59,3	57,1	65,2	55,6	< 0,001	49,7	57,7	63,7	59,7	65,7	60,3	< 0,001	46,0	52,2	60,1	57,6	65,0	56,5	< 0,001
Segundo trimestre	49,8	43,9	36,8	37,5	31,4	40,2		46,2	38,7	32,0	37,0	32,9	35,9		49,2	43,0	35,8	37,5	31,4	39,3	
Terceiro trimestre	5,0	4,1	3,9	5,4	3,4	4,2		4,1	3,6	4,3	3,3	1,4	3,8		4,8	4,8	4,1	4,9	3,6	4,2	
**Número de consultas de pré-natal** ^b^																					
De 1 a 3	17,7	14,1	9,0	9,9	8,5	11,7	< 0,001	15,1	8,4	5,7	6,9	4,3	7,4	< 0,001	17,2	13,1	8,3	9,2	7,7	10,9	< 0,001
De 4 a 5	28,1	27,0	14,0	17,3	21,2	20,4		24,6	23,0	16,0	15,4	15,9	18,6		27,5	26,3	14,4	16,9	20,3	20,0	
6 ou mais	54,2	58,9	77,0	72,8	70,3	67,9		60,3	68,6	78,3	77,7	79,8	74,0		55,3	60,6	77,3	73,9	72,0	69,1	
**Recebimento de cartão da gestante durante o pré-natal** ^b^																					
Não	0,7	1,1	1,4	0,8	2,4	1,2	0,001	0,5	1,6	0,5	0,6	0,9	0,9	0,043	0,7	1,2	1,1	0,8	2,1	1,1	0,002
Sim	99,3	98,9	98,6	99,2	97,6	98,8		99,5	98,4	99,5	99,4	99,1	99,1		99,3	98,8	98,9	99,2	97,9	98,9	
**Apresentou cartão da gestante na admissão para o parto** ^b^																					
Não	35,9	25,8	20,0	16,4	54,0	25,4	< 0,001	22,9	17,9	13,5	10,4	38,4	16,5	< 0,001	33,6	24,4	18,5	15,0	51,2	23,6	< 0,001
Sim	64,1	74,2	80,0	83,6	46,0	74,6		77,1	82,1	86,5	89,6	61,6	83,5		66,4	75,6	81,5	85,0	48,8	76,4	
**Glicemia** ^c^																					
Não	23,3	23,7	17,7	10,4	18,8	19,2	< 0,001	12,4	16,0	12,0	6,5	11,5	12,2	< 0,001	21,1	22,2	16,4	9,5	17,1	17,7	< 0,001
Sim, uma	49,3	54,4	45,7	40,0	52,7	48,2		48,6	49,1	40,4	33,5	43,1	42,5		49,2	53,4	44,4	38,4	50,5	47,0	
Sim, duas	27,4	21,9	36,6	49,5	28,6	32,6		39,0	34,9	47,7	60,0	45,4	45,3		29,7	24,4	39,2	52,1	32,4	35,4	
**Urina (EAS)** ^c^																					
Não	24,8	19,1	14,9	8,4	18,3	16,4	< 0,001	12,5	13,7	11,5	5,8	10,0	11,2	< 0,001	22,3	18,0	14,1	7,8	16,4	15,3	< 0,001
Sim	75,2	80,9	85,1	91,6	81,7	83,6		87,5	86,3	88,5	94,2	90,0	88,8		77,7	82,0	85,9	92,2	83,6	84,7	
**Ultrassonografia** ^c^																					
Não	32,9	18,9	9,6	9,2	14,3	14,8	< 0,001	27,0	15,8	8,2	6,7	8,5	11,6	< 0,001	31,7	18,3	9,3	8,6	13,0	14,1	< 0,001
Sim	67,1	81,1	90,4	90,8	85,7	85,2		73,0	84,2	91,8	93,3	91,5	88,4		68,3	81,7	90,7	91,4	87,0	85,9	
**Exame VDRL na gestação** ^c^																					
Não	22,8	17,7	9,5	5,5	17,2	13,1	< 0,001	14,6	11,5	7,4	4,5	9,3	8,7	< 0,001	21,1	16,5	9,3	5,3	15,4	12,1	< 0,001
Sim, um	50,0	54,2	46,9	38,7	45,8	48,3		53,7	50,5	44,0	34,2	45,0	45,2		50,7	53,2	46,0	37,6	45,6	47,7	
Sim, dois	27,2	28,1	43,6	55,8	37,0	38,6		31,7	38,0	48,6	61,3	45,7	46,1		28,2	30,3	44,7	57,1	39,0	40,2	
**Exame para HIV na gestação** ^c^																					
Não	31,6	35,5	14,3	8,1	19,6	21,9	< 0,001	28,8	28,4	11,5	6,9	15,4	16,8	< 0,001	31,0	34,1	13,7	7,8	18,6	20,7	< 0,001
Sim, um	50,5	51,3	53,4	49,8	47,9	51,8		52,8	51,5	55,4	48,0	48,4	52,8		51,0	51,4	53,8	49,4	48,1	52,1	
Sim, dois	17,9	13,2	32,3	42,1	32,5	26,3		18,4	20,1	33,1	45,1	36,2	30,4		18,0	14,5	32,5	42,8	33,3	27,2	
**Vinculação à maternidade** ^b^																					
Não	52,1	50,4	40,1	41,2	43,5	45,0	< 0,001	50,7	40,7	36,0	34,9	34,4	38,4	< 0,001	51,8	48,7	39,2	39,7	41,8	43,6	< 0,001
Sim	47,9	49,6	59,9	58,8	56,5	55,0		49,3	59,3	64,0	65,1	65,6	61,6		48,2	51,3	60,8	60,3	58,2	56,4	
**Parto na maternidade vinculada** ^d^																					
Não	14,2	23,3	19,7	8,7	16,0	18,5	< 0,001	14,5	25,0	17,9	10,4	19,0	18,4	< 0,001	14,2	23,7	19,3	9,0	16,6	18,5	< 0,001
Sim	85,8	76,7	80,3	91,3	84,0	81,5		85,5	75,0	82,1	89,6	81,0	81,6		85,8	76,3	80,7	91,0	83,4	81,5	
**Peregrinação para o parto**																					
Não	80,0	67,2	78,5	89,9	76,4	76,4	< 0,001	78,2	65,0	81,0	87,0	75,8	77,0	< 0,001	79,7	66,9	79,1	89,2	76,3	76,5	< 0,001
Sim, um hospital	17,3	27,7	17,2	9,2	20,1	19,7		19,6	29,6	16,0	12,2	21,9	19,8		17,7	28,0	16,9	9,9	20,4	19,7	
Sim, dois hospitais	2,7	5,1	4,3	0,9	3,5	3,9		2,2	5,4	3,0	0,8	2,3	3,2		2,6	5,1	4,0	0,9	3,3	3,8	
**Tipo de hospital do parto**																					
Público	71,8	58,6	42,8	28,9	56,5	50,3	< 0,001	83,0	67,1	48,1	35,6	63,2	55,7	< 0,001	73,5	60,1	43,9	30,4	57,5	51,3	< 0,001
Misto	28,2	41,4	57,2	71,1	43,5	49,7		17,0	32,9	51,9	64,4	36,8	44,3		26,5	39,9	56,1	69,6	42,5	48,7	
**Localidade do hospital do parto**																					
Capital	45,2	34,7	32,2	22,3	64,3	35,4	< 0,001	45,8	50,2	39,2	30,5	70,4	43,4	< 0,001	45,2	37,5	33,7	24,1	65,2	37,0	< 0,001
Não capital	54,8	65,3	67,8	77,7	35,7	64,6		54,2	49,8	60,8	69,5	29,6	56,6		54,8	62,5	66,3	75,9	34,8	63,0	

PSF: Programa Saúde da Família; UBS: unidade básica de saúde

^a^Teste do χ^2^.

^b^Incluídas na análise as mulheres que fizeram pré-natal.

^c^Incluídas na análise as mulheres que fizeram pré-natal e apresentaram o cartão de pré-natal na admissão para o parto.

^d^Incluídas na análise as mulheres que fizeram pré-natal, apresentaram o cartão de pré-natal na admissão para o parto e receberam orientação sobre a maternidade de referência para a assistência ao parto.

O recebimento de cartão da gestante cobriu quase a totalidade das mulheres. Entretanto, nem todas apresentaram o cartão na admissão para o parto. A cobertura no país de pelo menos um exame VDRL e um HIV na gestação foi de 88% e 79%, respectivamente, tendo as regiões Norte e Nordeste as menores prevalências. A cobertura dos exames de glicemia e EAS foi próxima a 85%, tendo a região Sul a maior prevalência. Com relação à ultrassonografia, a região Norte apresentou o maior déficit, com cobertura inferior a 70% ( Tabela 2 ).

Pouco mais da metade das mulheres foram vinculadas à maternidade durante o pré-natal. A região Sul se destacou com mais de 90% das mulheres com parto nas maternidades indicadas. A peregrinação foi enfrentada por mais de 20% das mulheres, atingindo mais de 30% no Nordeste ( Tabela 2 ).

As mulheres com intercorrências tiveram melhores indicadores de pré-natal que as mulheres sem intercorrências. Para as primeiras, foi observada maior prevalência de assistência pré-natal, de início precoce e de realização de seis ou mais consultas. A cobertura de exames VDRL e HIV também foi superior. No Nordeste a diferença observada entre as mulheres com ou sem intercorrências foi mais proeminente que nas outras regiões ( Tabela 2 ).

Com relação à adequação do pré-natal, que considerou tanto o início precoce quanto o número mínimo de consultas, as regiões Sudeste e Sul apresentaram as maiores prevalências. Já para a adequação global 1, a prevalência caiu substancialmente em todas as regiões. Considerando o critério mais restrito, adequação global 2, a prevalência no país foi de apenas 16%, tendo a região Nordeste o pior resultado, 10% ( Tabela 3 ). A região Centro-Oeste teve a maior taxa de cesariana (49,5%) e a Nordeste, a menor (41,6%). A Sudeste teve a menor taxa de cesariana anteparto (6,8%), conforme observado na Tabela 3 .

**Tabela 3:** Adequação do pré-natal e desfechos neonatais e maternos em mulheres com parto pago pelo Sistema Único de Saúde segundo risco materno e macrorregiões do Brasil, 2011–2012.

	Mulheres sem intercorrências							Mulheres com intercorrências							Todas as mulheres						
		
	N n = 1.773	NE n = 4.674	SE n = 6.143	S n = 1.727	CO n = 993	Brasil n = 15.310	P-valor^a^	N n = 1.773	NE n = 4.674	SE n = 6.143	S n = 1.727	CO n = 993	Brasil n = 15.310	P-valor^a^	N n = 1.773	NE n = 4.674	SE n = 6.143	S n = 1.727	CO n = 993	Brasil n = 15.310	P-valor^a^
																	
	%	%	%	%	%	%		%	%	%	%	%	%		%	%	%	%	%	%	
**Adequação do pré-natal** ^b^	47,3	52,6	67,1	65,8	63,4	60,1	< 0,001	55,5	64,5	72,0	69,5	72,6	68,2	< 0,001	48,7	54,7	68,2	66,7	65,0	61,7	< 0,001
**Adequação do pré-natal global 1** ^c^	10,1	11,4	21,6	24,6	18,5	17,7	< 0,001	12,0	21,0	29,4	29,4	26,9	25,6	< 0,001	10,5	13,3	23,4	25,7	20,4	19,4	< 0,001
**Adequação do pré-natal global 2** ^d^	9,6	8,8	17,3	22,5	15,1	14,5	< 0,001	11,7	16,2	24,0	26,1	24,8	21,2	< 0,001	10,0	10,2	18,9	23,4	17,3	16,0	< 0,001
**Tipo de parto**																					
Vaginal	59,1	63,0	62,0	59,8	57,2	61,4	< 0,001	36,2	37,2	38,8	45,3	20,6	37,9	< 0,001	55,1	58,4	57,0	56,5	50,5	56,7	< 0,001
Cesariana intraparto	31,7	27,2	31,4	32,0	33,7	30,4		52,6	50,6	53,4	45,3	68,2	52,4		35,3	31,4	36,2	35,1	40,0	34,8	
Cesariana anteparto	9,3	9,8	6,6	8,2	9,1	8,2		11,1	12,2	7,8	9,4	11,2	9,7		9,6	10,2	6,8	8,5	9,5	8,5	
**Desfechos**																					
Prematuridade espontânea	8,5	9,0	6,7	7,4	8,4	7,8	< 0,001	9,8	8,1	5,8	8,4	5,8	7,2	0,023	8,8	8,9	6,5	7,6	7,9	7,7	< 0,001
Prematuridade por intervenção	3,5	1,9	2,1	2,5	1,4	2,2	< 0,001	11,9	11,8	11,9	10,5	12,1	11,7	0,949	4,9	3,7	4,2	4,4	3,4	4,1	0,096
Baixo peso ao nascer (< 2.500 g)	7,9	8,9	7,6	7,7	7,8	8,1	0,124	17,9	18,7	13,5	12,7	17,6	15,4	0,001	9,6	10,7	8,9	8,9	9,6	9,5	0,001
CIUR (< percentil 10)	5,9	7,7	7,9	7,0	8,8	7,6	< 0,001	11,6	10,3	7,1	6,3	8,1	8,4	< 0,001	6,9	8,2	7,8	6,8	8,7	7,7	< 0,001
Apgar no 5º minuto < 8	1,6	3,4	1,9	1,3	1,2	2,2	< 0,001	3,2	3,0	2,5	1,8	2,9	2,6	0,615	1,9	3,3	2,0	1,4	1,5	2,3	< 0,001
*Near miss* neonatal	9,2	9,1	10,4	10,5	8,9	9,8	0,100	15,9	17,6	18,9	17,2	17,9	18,0	0,670	10,3	10,6	12,2	12,0	10,6	11,4	0,061
*Near miss* materno	0,5	0,3	0,3	0,6	0,2	0,4	0,318	3,4	5,5	3,3	3,3	7,6	4,1	0,004	1,0	1,2	1,0	1,2	1,6	1,1	0,333

CIUR: crescimento intrauterino restrito (< percentil 10 na curva Intergrowth)

^a^Teste do χ^2^.

^b^Incluídas na análise as mulheres que fizeram pré-natal. O pré-natal foi considerado adequado quando teve início até a 12ª semana gestacional e contou com número adequado (100%) de consultas para a idade gestacional no parto, considerando o calendário de seis consultas.

^c^Incluídas na análise as mulheres que fizeram pré-natal e apresentaram o cartão de pré-natal na admissão para o parto. O pré-natal foi considerado adequado quando teve início até a 12ª semana gestacional e contou com número adequado (100%) de consultas para a idade gestacional no parto, considerando o calendário de seis consultas, o registro de pelo menos um resultado de cada um dos exames preconizados na rotina de pré-natal e orientação sobre a maternidade de referência para a assistência ao parto.

^d^Incluídas na análise as mulheres que fizeram pré-natal, apresentaram o cartão de pré-natal na admissão para o parto e receberam orientação sobre a maternidade de referência para a assistência ao parto.

O pré-natal foi considerado adequado quando teve início até a 12ª semana gestacional; contou com número adequado (100%) de consultas para a idade gestacional no parto, considerando o calendário de seis consultas, o registro de pelo menos um resultado de cada um dos exames preconizados na rotina de pré-natal e orientação sobre a maternidade de referência para a assistência ao parto; e o parto foi na maternidade de referência para a assistência ao parto.

As regiões Norte, Nordeste e Centro-Oeste apresentaram os piores resultados neonatais, principalmente para prematuridade espontânea, BPN e CIUR. Foram encontradas mais desigualdades regionais e piores desfechos entre as mulheres com intercorrências ( Tabela 3 ).

A peregrinação para o parto se associou com todos os desfechos neonatais, exceto com CIUR, mesmo após o ajuste pelas variáveis de confusão. A razão de chances variou de 1,48 (IC95% 1,23–1,78) para o *near miss* neonatal a 1,62 (IC95% 1,27–2,06) para a prematuridade iniciada por intervenção. Entre as mulheres com alguma intercorrência, essa exposição se associou ainda mais fortemente à prematuridade iniciada por intervenção e Apgar no 5º min < 8, com OR de 1,98 (IC95% 1,49–2,65) e 2,19 (IC95% 1,31–3,68), respectivamente. A inadequação do pré-natal se associou à prematuridade espontânea nos dois grupos de mulheres ( Tabela 4 ).

**Tabela 4:** Desfechos neonatais e maternos associados à peregrinação e pré-natal inadequado em mulheres com parto pago pelo Sistema Único de Saúde segundo risco materno no Brasil, 2011–2012.

	Prematuridade espontânea	Prematuridade iniciada por intervenção	BPN	CIUR	Apgar 5 min < 8	Near miss neonatal	Near miss materno
						
	OR^a^(IC95%)	OR^a^(IC95%)	OR^a^(IC95%)	OR^a^(IC95%)	OR^a^(IC95%)	OR^a^(IC95%)	OR^a^(IC95%)
**Peregrinação**							
Todas as mulheres	1,61 (1,29–2,01)	1,62 (1,27–2,06)	1,56 (1,28–1,89)	1,19 (0,95–1,49)	1,56 (1,19–2,03)	1,48 (1,23–1,78)	1,38 (0,81–2,36)
Mulheres sem intercorrências	1,60 (1,25–2,04)	1,32 (0,92–1,91)	1,52 (1,23–1,87)	1,14 (0,86–1,51)	1,41 (1,03–1,94)	1,50 (1,21–1,85)	1,34 (0,70–2,56)
Mulheres com intercorrências	1,67 (1,06–2,61)	1,98 (1,49–2,65)	1,69 (1,23–2,31)	1,37 (1,03–1,83)	2,19 (1,31–3,68)	1,45 (1,16–1,81)	1,39 (0,70–2,77)
**Pré-natal inadequado (início e consultas)**							
Todas as mulheres	1,51 (1,24–1,83)	1,06 (0,73–1,55)	1,25 (0,99–1,56)	1,11 (0,88–1,38)	0,96 (0,68–1,36)	1,14 (0,95–1,37)	0,96 (0,54–1,68)
Mulheres sem intercorrências	1,47 (1,18–1,82)	0,95 (0,62–1,46)	1,21 (0,94–1,54)	1,16 (0,90–1,51)	1,00 (0,69–1,43)	1,08 (0,88–1,34)	0,82 (0,29–2,36)
Mulheres com intercorrências	1,73 (1,02–2,94)	1,14 (0,66–1,97)	1,32 (0,90–1,92)	0,94 (0,59–1,50)	0,81 (0,39–1,71)	1,28 (0,85–1,93)	0,97 (0,55–1,73)

OR: *odds ratio* (razão de chances); IC95%: intervalo de confiança de 95%; BPN: baixo peso ao nascer (< 2.500 g); CIUR: crescimento intrauterino restrito (< percentil 10 na curva Intergrowth).

^a^Ajustada por região, idade, escolaridade e paridade.

^b^Ajustada por região, idade, escolaridade, paridade e tipo de parto.

## DISCUSSÃO

É quase universal a cobertura da atenção pré-natal no Brasil para as gestantes que utilizam o SUS, pelo critério de pelo menos uma consulta, mas esse panorama vai se modificando à medida que se consideram outros parâmetros. Ao incluir recomendações como o número mínimo de exames realizados e vinculação à maternidade para o parto, a adequação se reduz para pouco mais de um quarto de todas as mulheres, diminuindo ainda mais se considerarmos a vinculação efetiva a uma maternidade.

Tomasi et al.^15^analisaram a assistência pré-natal no SUS no mesmo período deste estudo e observaram que 89% das gestantes fizeram seis ou mais consultas, mas apenas 15% das entrevistadas receberam atenção pré-natal adequada. As diferenças nos percentuais de cobertura entre os dois estudos se devem ao fato de que, no estudo de Tomasi, foram obtidas as informações de mulheres atendidas pelas equipes de unidades básicas de saúde (UBS), com 18 anos ou mais de idade, presentes nas unidades por ocasião da avaliação; enquanto o Nascer no Brasil entrevistou uma amostra representativa de mulheres no momento do parto, já que 99% dos partos ocorreram em hospitais, tendo assim um panorama mais aproximado da real cobertura pré-natal. Além disso, o critério utilizado para adequação do pré-natal foi diferente nas duas pesquisas, o que também contribui para as diferenças encontradas.

Os achados mostram que o SUS ainda apresenta falhas na continuidade e qualidade da atenção dispensada. Monteiro et al.^16^verificaram, no estado de São Paulo, que o acesso quase universal aos serviços de assistência à saúde apresentava problemas na qualidade vivenciados principalmente pelos grupos socioeconômicos mais baixos, a maioria dos quais utilizavam o SUS.

As desigualdades sociais e econômicas entre as regiões geográficas do país são evidentes neste estudo. Na esfera da saúde reprodutiva, nas regiões mais desfavorecidas as mulheres são mais jovens, têm maior proporção de gravidez na adolescência e maior paridade. Por serem mais velhas e terem acesso a melhor diagnóstico clínico, as mulheres das regiões Sul e Sudeste apresentaram maiores proporções de intercorrências clínicas. No entanto, foi o Norte que apresentou a mesma proporção de mulheres sem assistência pré-natal para o grupo com e sem ocorrência obstétrica. Esse pior desempenho da região pode estar associado às dificuldades geográficas, grandes distâncias e barreiras de acesso aos grandes centros para diagnósticos e tratamento, ausência de profissionais qualificados etc. É também maior nessa região a proporção de partos domiciliares, que, quando realizados por profissionais não qualificados, associam-se com maior taxa de mortalidade infantil^17^. Viellas et al.^18^, estudando a atenção pré-natal no Brasil, identificaram que a menor cobertura e início tardio nas regiões Norte e Nordeste em gestantes com menor escolaridade estavam associadas mais a barreiras de acesso do que ao desconhecimento da gravidez e a problemas de natureza pessoal da gestante.

Neste estudo, gestantes com pré-natal inadequado foram mais suscetíveis a terem recém-nascidos prematuros espontaneamente. Um estudo com essa mesma amostra de mulheres observou que a prematuridade espontânea se associou com a pobreza e inadequação do pré-natal^19^, fatores que contribuem para a manutenção das altas taxas da mortalidade infantil do país^20^, dado que o nascimento pré-termo é o maior fator de risco para a morbidade e mortalidade no primeiro ano de vida e na infância^21^.

Outra questão identificada é a desconexão entre os níveis de atenção ambulatorial e hospitalar, aspecto que se reveste de importância, uma vez que conhecer a maternidade contribui para o bem-estar da mulher e o bom andamento do TP^22^. A lei da vinculação à maternidade já completou dez anos, mas não foi ainda devidamente implementada no país. Chama a atenção que a região Sul conseguiu vinculação efetiva para mais de 90% das gestantes, evidenciando uma maior organização do sistema e melhor coordenação do cuidado, continuidade e hierarquização das ações na saúde materno-infantil.

Foi elevada a taxa de cesariana em todas as regiões, principalmente para as mulheres com intercorrências obstétricas, 75% maior do que no grupo de baixo risco. A taxa média de 30,4% de cesariana no grupo de baixo risco se aproxima do valor da taxa global dos EUA e está muito acima da taxa dos países europeus, que ficam em torno de 20 a 25%^23^. A região Sudeste, que teve a menor taxa de cesariana intraparto, teve também a menor taxa de cesariana do setor público próprio do SUS (dados não mostrados). Nos países europeus a maior proporção de cesarianas é intraparto, diferentemente do que ocorre no Brasil, onde predomina a anteparto ou cesariana agendada^24^. Esse melhor resultado da região Sudeste pode ser em consequência do movimento em curso de mudança no modelo de atenção ao parto nos hospitais próprios do SUS dessa região, focado nas melhores evidências científicas, sem desconsiderar o movimento das mulheres na busca por uma atenção ao parto menos medicalizada.

As mulheres que tiveram ocorrências obstétricas receberam melhor atenção pré-natal que as de baixo risco. Isso mostra que a atenção básica vem tendo alguma efetividade em identificar esses problemas e atender melhor às gestantes de risco. No entanto, apesar desse esforço, o sistema falhou no processo de integralidade do atendimento, não dando continuidade no acesso à maternidade.

A falha do sistema na coordenação e integralidade do cuidado no momento do parto, com peregrinação de muitas mulheres, associou-se a grandes prejuízos para os recém-nascidos. Na obstetrícia é conhecido o papel que exercem as demoras no atendimento. Na década de 1990, autores ingleses propuseram um modelo teórico de três demoras conhecido como *three delays model* que as classifica em: fase I, a demora na decisão de procurar cuidados; fase II, a demora em chegar a uma unidade de cuidados adequados; e fase III, a demora em receber os cuidados adequados na instituição de referência^25^. Quando uma mulher peregrina por mais de um serviço de saúde, certamente estamos diante da fase II e III. Pacagnella et al.^26^, utilizando esses conceitos, analisaram o papel das demoras na morbidade materna grave no Brasil e encontrou associação entre esse desfecho e as demoras no atendimento. Na presente análise, a peregrinação para o parto se associou a quase todos os desfechos negativos no recém-nascido, principalmente no grupo de gestantes com intercorrências, possivelmente por demandarem mais intervenções clínicas.

Uma recente análise com dados do Nascer no Brasil mostrou que, no SUS, 32% das mulheres de risco obstétrico foram atendidas em hospitais sem unidades de cuidado intensivo, enquanto 29,5% das de baixo risco realizaram o parto em hospitais com esse tipo de recurso^27^. Isso mostra que o sistema não está adequadamente articulado para disponibilizar atenção de alta complexidade a quem necessita, ofertando-a desnecessariamente a quem não precisa. As consequências dessa desarticulação podem ser vistas neste estudo e merecem atenção dos gestores para evitar sofrimento, complicações e abreviamento de vidas.

O pré-natal é uma ação programática típica da atenção primária, e os resultados do estudo comprovam esse fato e sua relação com os resultados obstétricos. Uma vez que 90% das entrevistadas fizeram seu pré-natal na rede básica de saúde, as ações de qualificação das equipes e dos processos de trabalho têm papel fundamental na melhoria dos cuidados ao bebê e à gestante. Fachini et al.^28^destacam a importância no aumento da efetividade da Estratégia Saúde da Família, considerando seu efeito mediador na atenção à saúde.

Em conclusão, persistem as desigualdades regionais, as barreiras no acesso e a inadequação da atenção pré-natal, contribuindo para resultados adversos para os recém-nascidos. A melhoria da qualidade da atenção pré-natal e a coordenação e integralidade do cuidado no momento do parto têm potencial impacto sobre as taxas de prematuridade e consequentemente sobre a redução da taxa de morbimortalidade infantil no país.
